# Linkage disequilibrium patterns and persistence of phase in purebred and crossbred pig (*Sus scrofa*) populations

**DOI:** 10.1186/s12863-014-0126-3

**Published:** 2014-11-25

**Authors:** Renata Veroneze, John WM Bastiaansen, Egbert F Knol, Simone EF Guimarães, Fabyano F Silva, Barbara Harlizius, Marcos S Lopes, Paulo S Lopes

**Affiliations:** Departamento de Zootecnia, Universidade Federal de Viçosa, Av. PH Holfs, Viçosa, 36570-000 MG Brazil; Animal Breeding and Genomics Centre, Wageningen University, Droevendaalsesteeg 1, Wageningen, 6708 PB The Netherlands; Topigs Norsvin, PO Box 43, Beuningen, 6640 AA The Netherlands

**Keywords:** Nonlinear model, Single nucleotide polymorphism, SNP, Genomic selection

## Abstract

**Background:**

Genomic selection and genomic wide association studies are widely used methods that aim to exploit the linkage disequilibrium (LD) between markers and quantitative trait loci (QTL). Securing a sufficiently large set of genotypes and phenotypes can be a limiting factor that may be overcome by combining data from multiple breeds or using crossbred information. However, the estimated effect of a marker in one breed or a crossbred can only be useful for the selection of animals in another breed if there is a correspondence of the phase between the marker and the QTL across breeds. Using data of five pure pig (*Sus scrofa*) lines (SL1, SL2, SL3, DL1, DL2), one F_1_ cross (DLF1) and two commercial finishing crosses (TER1 and TER2), the objectives of this study were: (i) to compare the equality of LD decay curves of different pig populations; and (ii) to evaluate the persistence of the LD phase across lines or final crosses.

**Results:**

Almost all of the lines presented different extents of LD, except for the SL2 and DL3, both of which exhibited the same extent of LD. Similar levels of LD over large distances were found in crossbred and pure lines. The crossbred animals (DLF1, TER1 and TER2) presented a high persistence of phase with their parental lines, suggesting that the available porcine single nucleotide polymorphism (SNP) chip should be dense enough to include markers that have the same LD phase with QTL across crossbred and parental pure lines. The persistence of phase across pure lines varied considerably between the different line comparisons; however, correlations were above 0.8 for all line comparisons when marker distances were smaller than 50 kb.

**Conclusions:**

This study showed that crossbred populations could be very useful as a reference for the selection of pure lines by means of the available SNP chip panel. Here, we also pinpoint pure lines that could be combined in a *multiline* training population. However, if *multiline* reference populations are used for genomic selection, the required density of SNP panels should be higher compared with a single breed reference population.

**Electronic supplementary material:**

The online version of this article (doi:10.1186/s12863-014-0126-3) contains supplementary material, which is available to authorized users.

## Background

Linkage disequilibrium (LD) is a nonrandom association between alleles at different loci [1]. There has been a growing interest in LD analysis with the explosion of genomic selection (GS) and genome wide association studies (GWAS) published in recent years. Both GS and GWAS exploit the LD between markers and quantitative trait loci (QTL) to estimate genomic breeding values (GEBV) or to detect regions that control traits of interest.

The accuracy of GEBV depends on the LD between the markers and the QTL, the number of animals in the reference population, the heritability of the trait, the distribution of QTL effects [2] and the level of family relationship between the reference population and the selection candidates [3]. The number of animals in the reference population is a critical parameter for the accuracy of GS [4], and this value can limit the application of GS in certain situations. This constraint may be overcome by increasing the reference population size by combining animals from different breeds or lines [5]. Daetwyler et al. [6] showed that GEBV are more accurate than pedigree-based best linear unbiased prediction (BLUP) using a multibreed sheep training population.

Another approach that can be used to acquire a larger reference population is the inclusion of crossbred animal information, because large populations are available in commercial farms. Using crossbreds has several advantages: one crossbred population could be used to select more than one pure line, the phenotypes of production animals can be more relevant for breeders and the animals can be selected for traits that are not measured in the nucleus herd (e.g. disease resistance). In addition, using crossbred data it may be possible to account for heterotic effects in the selection. Using marker information, Amuzu-Aweh et al. [7] showed that it was possible to identify specific sires whose offspring could be expected to show higher levels of heterosis. These approaches are especially attractive for the pig industry, where breeding companies keep a range of sire and dam lines. Using crossbred reference populations could reduce the need to establish separate large reference populations for each pure line.

To evaluate the potential for using a reference population from a different breed or cross, it is essential to know the LD in those breeds and crosses, as well as the persistence of the LD phase across these populations and with the population of selection candidates. Assuming that QTL effects are the same in different breeds, the estimated effect of a marker in one breed can still only be used to select animals in another breed if the phase of the marker and QTL alleles are the same in both breeds [8]. GS uses direct relationships and LD to predict breeding values. When predictions are carried out in populations with distantly related individuals, the accuracy is mainly determined by LD between markers and QTL, while predictions with closely related individuals rely mainly on direct relationships [9]. Thus, when the relatedness across breeds is small, the accuracy of prediction is mainly reflected in the LD between markers and QTL. In addition, knowledge of the persistence of phase across physical distance between markers for two populations can be used to determine which marker density is needed to provide the same LD phase across these populations [10].

Badke et al. [11], when evaluating the Landrace, Yorkshire, Hampshire and Duroc breeds, found that the correlation of phase ranged between 0.87 for Duroc-Yorkshire and 0.92 for Landrace-Yorkshire, for markers with a pairwise distance <10 Kb. While, for the same distance, Wang et al. [12] found a persistence of phase of 0.61 for Duroc-Landrace, 0.57 for Duroc-Yorkshire and 0.66 for Landrace-Yorkshire. Studies evaluating LD and persistence of phase in crossbred pig lines are scarce, and the comparison of LD decay in different populations has been achieved visually using average LD [10-14], without the application of models or statistical comparisons.

In the present study, we evaluated five pig pure lines (SL1, SL2, SL3, DL1, DL2), one F_1_ cross (DLF1) and two commercial finishing crosses (TER1 and TER2) representing the crossbred structure of pork production. The objectives of this study were: (i) to compare the equality of LD decay curves of different populations; and (ii) to evaluate the persistence of phase across populations.

## Results

### LD decay

The nonlinear model for the decay of LD with distance was adjusted to simultaneously describe multiple lines. The model parameter *β*_*k*_ describes the decline of LD with distance for each line. The estimates of ${\widehat{\beta}}_k$ ranged from 1.25 × 10^−3^ to 2.92 × 10^−3^ and were all significantly different from zero (p-value <0.01) (Table 1).

**Table 1:** **Parameter estimate (** ${\overset{\frown }{\beta}}_k$ **), standard error and p-value for the nonlinear fitted model for each line**

**Line**	${\overset{\frown }{\boldsymbol{\upbeta}}}_{\mathbf{k}}$	**Std. Error**	**p-value**
SL1	1.78 × 10^−3^	4.76 × 10^−6^	<10^−3^
SL2	1.25 × 10^−3^	2.89 × 10^−6^	<10^−3^
SL3	1.69 × 10^−3^	4.42 × 10^−6^	<10^−3^
DL1	2.12 × 10^−3^	6.09 × 10^−6^	<10^−3^
DL2	1.71 × 10^−3^	4.49 × 10^−6^	<10^−3^
DLF1	2.44 × 10^−3^	7.46 × 10^−6^	<10^−3^
TER1	2.92 × 10^−3^	9.63 × 10^−6^	<10^−3^
TER2	2.03 × 10^−3^	5.75 × 10^−6^	<10^−3^

The adjusted model to describe the LD permits a statistical comparison of the lines with respect to the decline of LD with distance, which is important to infer the size of single nucleotide polymorphism (SNP) panels for GS and GWAS in these lines. To compare the lines, the equality of the LD curves was tested. The first hypothesis tested $\left({H}_0^{(1)}:{\upbeta}_{\mathrm{k}}=\upbeta\ \forall\ k\right)$ states that the model to describe the LD decay is the same for all lines. This hypothesis was rejected (p-value <10^−3^), which implies that at least one parameter β differs from the other parameters. Next, a pairwise comparison was carried out that aimed to identify which lines are equal or different regarding the parameter β. All pairwise comparisons were significantly different [see Additional file 1: Table S1], with the exception of the comparison between β_SL3_ and β_DL2_ (p-value 0.0117 > Bonferroni corrected significance α*). These results suggested that the same model could be used to describe the LD decay of these two lines. In addition, SL2 showed the smallest β value, which implied that this line has the largest extent of LD, while TER1 showed the largest β value and consequently the shortest LD.

The test of the equality of the LD decay curves showed that the overall pattern of LD decay differed between lines. The predicted LD was reported at specific marker distances (Table 2), with the highest values of predicted LD observed for SL2 at various distances, while TER1 presented the lowest values. SL3 and DL2 presented the same values of predicted LD, because the β parameters of these lines did not differ statistically. All lines presented low values of LD for marker distances above 3000 kb. At these large marker distances, the crossbreds exhibited similar levels of LD compared with the pure lines.

**Table 2:** **Predicted r** ^**2**^ **at various distances (Kb) for eight pig populations**

**Distance (Kb)**	**50**	**250**	**500**	**1000**	**2000**	**3000**
SL1	0.74	0.36	0.22	0.12	0.07	0.04
SL2	0.80	0.44	0.28	0.17	0.09	0.06
SL3	0.75	0.37	0.23	0.13	0.07	0.05
DL1	0.70	0.32	0.19	0.11	0.06	0.04
DL2	0.75	0.37	0.23	0.13	0.07	0.05
DLF1	0.67	0.29	0.17	0.09	0.05	0.03
TER1	0.63	0.26	0.15	0.08	0.04	0.03
TER2	0.71	0.33	0.20	0.11	0.06	0.04

Most of the studies on LD presented the average *r*^*2*^ at various distances to compare populations. To facilitate comparison with other studies and also to make a comparison with the predicted LD, the average and standard deviation of LD at various distances are shown in Table 3. The standard deviation of r^2^ tended to decrease when the distance between markers increased in all lines, which is expected, because at short distances the r^2^ values are much more variable. The average LD for markers less than 50 Kb apart ranged from 0.55 for SL2 to 0.46 for TER1, both of which are smaller than the predicted LD at the same marker distance. Similar to the predicted LD, SL2 presented the highest values of average LD at various distances, thus showing the same tendency for predicted and average values. However, the predicted LD was higher than the average for short distances (>50 Kb) and smaller for the largest distances (3000–3050 Kb) for all lines.

**Table 3:** **Average and standard deviation r** ^**2**^ **at various distances (Kb) for eight pig populations**

**Dist**	**0–50**	**200–250**	**500–550**	**1000–1050**	**2000–2050**	**3000–3050**
SL1	0.49 ± 0.37	0.30 ± 0.31	0.23 ± 0.27	0.18 ± 0.23	0.12 ± 0.19	0.10 ± 0.16
SL2	0.55 ± 0.37	0.35 ± 0.33	0.28 ± 0.30	0.21 ± 0.25	0.14 ± 0.21	0.11 ± 0.18
SL3	0.50 ± 0.37	0.29 ± 0.30	0.24 ± 0.27	0.18 ± 0.23	0.13 ± 0.19	0.10 ± 0.17
DL1	0.49 ± 0.36	0.29 ± 0.30	0.21 ± 0.26	0.16 ± 0.22	0.11 ± 0.18	0.09 ± 0.16
DL2	0.51 ± 0.37	0.31 ± 0.31	0.24 ± 0.27	0.18 ± 0.24	0.12 ± 0.19	0.09 ± 0.16
DLF1	0.47 ± 0.36	0.27 ± 0.29	0.20 ± 0.24	0.15 ± 0.21	0.10 ± 0.16	0.08 ± 0.14
TER1	0.46 ± 0.35	0.25 ± 0.28	0.18 ± 0.23	0.14 ± 0.19	0.09 ± 0.15	0.07 ± 0.13
TER2	0.50 ± 0.35	0.29 ± 0.29	0.22 ± 0.26	0.16 ± 0.22	0.11 ± 0.17	0.08 ± 0.15

### Persistence of linkage disequilibrium phase

In pig production, crossbred animals are used for reproduction on commercial farms. The line DLF1 represents these crossbred females, and crossing the dam lines DL1 and DL2 produces these animals. DLF1 presented a similar LD compared with lines DL1 and DL2, with high persistence of phase, a correlation of >0.9 for marker distances up to 150 Kb and a correlation of >0.8 for marker distances up to 1200 Kb.

**Figure 1:**
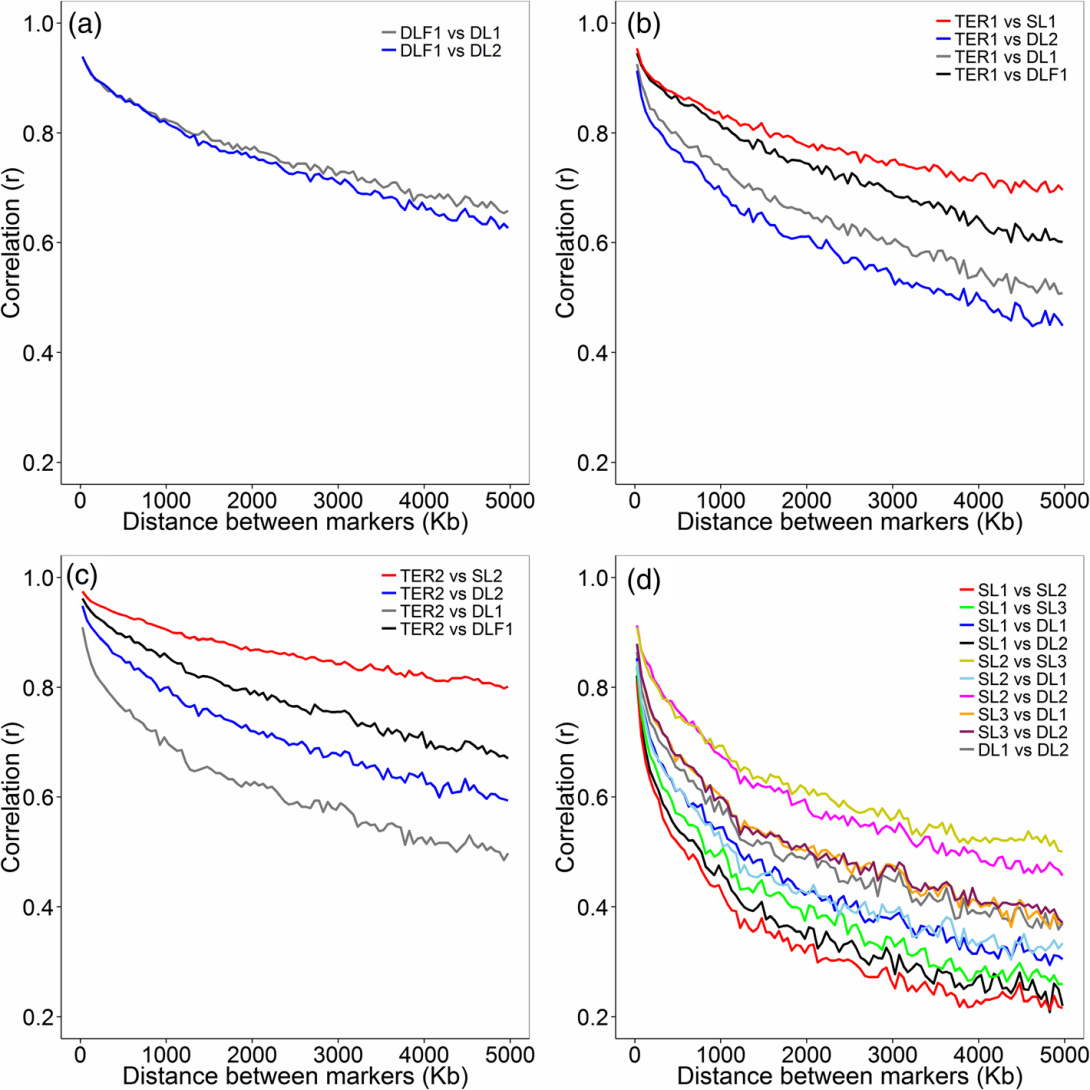
**Correlation of phase (** ***r***_***ij***_ **) in relation to the distance. a**. Correlation between F1 (DLF1) and its parental lines (DL1 and DL2). **b**. Correlation between terminal cross (TER1) and its (grand) parental lines (SL1, DLF1, DL1 and DL2). **c**. Correlation between terminal cross (TER2) and its (grand) parental lines (SL2, DLF1, DL1 and DL2). **d**. Correlation across all pure lines (SL1, SL2, SL3, DL1, DL2).

Commercial finishing pigs TER1 and TER2 are the end product of the pig industry, and are based on a cross between DLF1 and either SL1 or SL2, respectively. TER1 showed higher persistence of phase with SL1 and DLF1 compared with lines DL1 and DL2 (Figure 1b); this result was expected because the haplotype sharing is different between TER1 and these four populations. TER1 showed a correlation of phase of >0.9 for markers at distances below 200 Kb in relation to lines SL1, DLF1, DL1 and DL2 (Figure 1b).

Similar to TER1, TER2 showed greater persistence of phase with SL2 and DLF1 compared with lines DL1 and DL2 (Figure 1c). The distance at which the correlation of phase remained >0.9 was higher for TER2 compared with TER1, with distances of 1050 Kb, 400 Kb, 150 Kb and 50 Kb in relation to the lines SL2, DLF1, DL1 and DL2, respectively (Figure 1c).

Interestingly, for TER1, a higher persistence of phase was observed with DL1 than with DL2 (Figure 1b), but the reverse was observed for TER2, with a higher persistence of phase with DL2 than with DL1 (Figure 1c). These results can be explained by the contributions of different breeds to the different lines. SL1 and DL1 have contributions from the Landrace breed, while SL2 and DL2 have contributions from the Large White breed.

Persistence of phase across pure lines was evaluated to provide information towards the use of a *multiline* reference population for GS. The highest persistence of phase was observed between SL2 and SL3, and between SL2 and DL2, which exhibited a correlation of >0.9 for markers at distances up to 50 kb, and the persistence remained high at larger distances (Figure 1d). The lowest correlation was observed between SL1 and SL2 (0.81) for markers at distances up to 50 kb. Persistence of phase showed a considerable variation between the different line comparisons; however, correlations were above 0.8 for all line comparisons when marker distances were smaller than 50 kb. Common breeds in the line genetic background resulted in a higher persistence of phase. For multiline reference populations, a SNP panel denser than the currently available is necessary to keep the same phase across pure lines.

## Discussion

Using the equality of curves test, the LD decay was found to differ significantly for all except one of the pairwise comparisons between the pig lines. Persistence of phase was found to be highest between pure lines, especially for short distances below 50 kb. The persistence of LD between crossbreds and their (grand) parental lines followed the expectations based on the contributions that the different breeds made to each of the lines.

### Equality of LD curves

A formal comparison of the level of LD decay was made possible by our adjustments to the nonlinear model described by Sved (1971) [15]. All of the lines studied followed the same pattern of a rapid decrease in LD as the distance increased. Previous comparisons of LD decay between breeds or lines was performed using average r^2^ in distance bins [10,11,13,14] and/or adjusting a linear model to test the breed effect [16,17]. The equality of curves test permits not only the identification of the existence of line differences, but also allows for a pairwise comparison across all lines. The test revealed that six of the eight evaluated lines differ with respect to LD decay. Only the comparison between SL3 and DL2 was not rejected, which implied that the decrease in LD with the distance is the same for these two lines. The extent of LD provides an insight into the number of SNPs required for GS and GWAS. Lines SL3 and DL2 presented the same predicted LD; therefore, an identical marker density could be used for genomic studies in both lines. However, this does not imply that the same marker set is suitable in both lines, because different markers may be segregating in different lines. The test also revealed different extents of LD for six of the evaluated lines, with a higher LD observed for SL2 and a lower LD for TER1. This information implied that different marker densities should be used for GS and GWAS for these lines, which could also influence the accuracy of GS.

### LD in crossbreds

According to Reich et al. [18], the extent of LD depends on the number of generations that have passed since the occurrence of an LD-generating event. In crossbred populations, LD comprises the existing LD in the parent populations and new LD generated in the cross as a result of different allele frequencies in the parental breeds [19]. The average LD for markers at distances up to 50 kb ranged from 0.47 to 0.50 in crossbreds and from 0.49 to 0.55 in the pure lines, while for markers at distances between 3000 and 3050 Kb, the LD ranged from 0.07 to 0.08 in crossbreds and from 0.09 to 0.11 in the pure lines. Surprisingly, the LD over large distances was not higher in crossbreds. A possible explanation for these similar LD levels in crossbred and pure lines may be the similarities in allele frequencies, or in LD phase, between the (grand) parental lines of the crossbreds. With similar frequencies, limited LD is created because of crossing [19]. Similarity in the allele frequencies could be caused by the fact that the minor allele frequency (MAF) was one of the criteria used to select markers for the 60K beadchip, which may have reduced the differences in allele frequency across lines [20].

### LD in pigs from the literature

By evaluating LD in Finnish Landrace and Finnish Yorkshire pigs, Uimari and Tapio [13] found an average *r*^*2*^ of 0.47 and 0.49 for markers 30 kb apart, and these results are similar to our findings for DL1 (Landrace based line) and DL2 (Large White based line). In addition, Uimari and Tapio [13] reported *r*^*2*^ values of 0.09 and 0.12 for SNPs that were 5 Mb apart in Finnish Landrace and Finnish Yorkshire pigs, respectively, which is higher than the average *r*^*2*^ of 0.06 for DL1 and DL2 found in the present study.

By studying Duroc, Hampshire, Landrace and Large White from the USA, Badke et al. [11] detected average *r*^*2*^ values of 0.26, 0.25, 0.19 and 0.21 for SNPs that were 500 Kb apart, respectively, while in the present work, the lines SL1 (a combination of Duroc and Belgian Landrace), DL1 and DL2 presented average LDs of 0.23, 0.21 and 0.24, respectively. The differences regarding Duroc and SL1 could be explained by the breed composition of SL1, which contains Landrace genes, while differences in population structure, such as inbreeding and effective population size, could explain the LD differences of the Landrace and Large White breeds evaluated by Badke et al*.* [11] and between DL1 and DL2. At large distances (5 Mb), the LD levels were similar to those found by Badke et al. [11].

Evaluating the LD in Danish Landrace, Large White and Duroc, Wang et al. [12] found average LDs of 0.32, 0.32 and 0.35 for markers at a distance of 500 Kb, respectively, and these values are much higher than the values found in the present paper for DL1, DL2 and SL1 (0.21, 0.24 and 0.23, respectively). Parameters that are specific for a population, such as the inbreeding, effective population size and selection, can also result in different LD levels across populations. Studying the LD of local Spanish and Portuguese pig breeds and of wild pig populations, Herrero-Medrano et al. [21] found that the decay of LD was greater in wild boars than in the domestic breeds. Evaluating the LD of Chinese and Western pigs, Ai et al. [22] found that Chinese breeds have lower extents of LD than Western pigs.

### Implications for GS

An average LD greater than 0.2 has been reported to be required for GS [23], and this LD level was observed for most of the evaluated lines at marker distances between 500 and 550 kb. All lines exhibited an average r^2^ higher than 0.3 for markers 100–150 kb apart. Qanbari et al. [24] found an average r^2^ = 0.30 for markers at distances <25 kb for German Holstein cattle, and Bohmanova et al. [25] found r^2^ > 0.3 for markers at distances of 60 kb in American Holstein cattle. Thus, in agreement with Veroneze et al. [14] and Badke et al. [11], it seems that LD extends further in European commercial pig breeds than in Holstein cattle, which implies that the use of less dense SNP panels is possible for GWAS and GS in pigs. Evaluating the use of low density panels associated with genotype imputation in pig sire lines, Wellmann et al. [26] recommended that a panel with 384 markers could be used for genotyping selection candidates if at least one parent was genotyped at high-density. However, if multibreed reference populations are used for GS, the required density of SNP panels should be higher compared with a single breed reference population.

Persistence of phase is essential for the success of across lines GS. In the present paper, the persistence of LD phase was evaluated for eight commercial pig populations, thus representing the crossbreeding structure of pig production design.

The high persistence of phase for SNPs with a 150 Kb distance when comparing DLF1, DL1 and DL2 implies that similar marker effects may be expected across the evaluated lines. The available porcine SNP chip should be dense enough to include markers that have the same LD phase with QTL across DLF1, DL1 and DL2. The persistence of phase with DLF1 shows the potential use of an F_1_ commercial cross as a reference population to select purebred lines. However, using pig purebreds to predict crossbred performance, Hidalgo (personal communication) found that the accuracies of the breeding values are trait-dependent, which challenges the use of the crossbred information in breeding programs.

In a simulation study using a crossbred (F_1_) as the reference population to select purebred animals, Toosi et al. [19] found that using 10 markers per cM (a density approximately equal to the present work) resulted in an accuracy of GEBV of 0.78, while training in the same breed as the validation population resulted in a accuracy of 0.83. The authors concluded that crossbreds could be used to select purebreds without significant loss of accuracy. Crossbred animals can also be used as a source of information for genotype imputation, because of the high persistence of phase. Evaluating multi-breed imputations in Canadian dairy cattle breeds, Larmer et al. [27] found that multi-breed populations resulted in increased imputation accuracy for the breeds Guernsey and Ayrshire, where consistency of gametic phase was high.

Using crossbred animals in the reference population is expected to have a number of advantages. First, the utilization of crossbred performance to select purebreds enables selection for traits that cannot be measured at nucleus farms, such as disease resistance [28]. Second, a crossbred reference population may allow for reduced costs of GS when the same crossbred performance can be used as information for selection in two or more pure lines. Third, the use of crossbreds permits exploitation of the heterotic effects, which cannot be done when the selection is performed exclusively in purebreds. However, for the use of crossbred information, pig breeding programs need to adapt their data collection to obtain the phenotypes of F_1_ sows and finishing pigs, which can be challenging, because these animals are held on commercial farms.

### Interpretation of the correlations between lines

The higher correlation of the LD phase in TER1 with SL1 and DLF1 compared with the correlation of TER1 with DL1 and DL2 was expected because the persistence of the LD phase tended to decrease when a smaller proportion of the genome is shared. TER1 shares 50% with both SL1 and DLF1, and only 25% with both DL1 and DL2. TER2 showed the same tendencies, showing a higher correlation with its parent lines, SL2 and DLF1, than with its grandparent lines, DL1 and DL2. The correlation of phase with TER2 was higher over much longer distances between markers compared with TER1. Correlations above 0.9 were observed for the LD between markers at distances up to 1050 kb and 400 kb when comparing TER2 with SL2 and DLF1. Our assumption was that the higher persistence of phase of TER2 with its paternal line SL2 is caused by the higher LD observed in SL2.

With the marker density provided by the pig 60K SNP panel, the data from TER1 could be used in GS strategies for SL1. Similarly, the data from TER2 could be used to select in SL2. The 60K SNP panel provides a marker density that shows a high persistence of LD phase between these lines. A much higher marker density would be necessary to ensure a persistence of phase between the lines TER1 and TER2 and between the dam lines DL1 and DL2.

While the correlations between crossbreds and their parental lines should allow for GS with a crossbred reference population using the SNP60Beadchip, the question remains whether the correlation of phase between pure lines is also high enough for a multibreed reference population design. The persistence of phase between pure lines depends on the time since their divergence took place [10]; i.e., the consistency of LD is directly related to the degree of relationship between lines [29]. The highest persistence of phase was observed between SL2 *vs*. SL3 and SL2 *vs*. DL2. As described in the Methods section, SL2 is a synthetic line resulting from the combination of the Large White and Pietrain breeds. SL3 is a Pietrain pure sire line and DL2 is a Large White pure dam line. Thus, the higher persistence of phase observed between SL2 *vs*. SL3 and SL2 *vs*. DL2 could be explained by the common breeds in the composition of these lines.

The persistence of phase of Duroc, Hampshire, Landrace and Large White breeds was studied by Badke et al. [11]. A correlation of phase of 0.92 was found between the breeds Landrace and Large White for markers at distances of 10 kb, which is similar to the correlation observed between the lines DL1 and DL2 (which are Landrace and Large White derived lines, respectively) for markers at the same distance (0.93). The persistences of phase between SL1 *vs*. DL1 and SL1 *vs*. DL2 were higher (0.92 and 0.90, respectively) than the values found by Badke et al. [11] between Duroc *vs*. Large White and Duroc *vs*. Landrace (0.87 for both) for markers at distances of 10 kb. Some difference was expected, because SL1 is a synthetic line of Duroc (mostly) and Landrace, so the highest persistence of phase in relation to the study of Badke et al. [11] could be caused by the presence of the Landrace breed in SL1.

The lowest correlations of phase were observed between all lines and SL1. By evaluating the persistence of phase in Landrace, Large White and Duroc, Wang et al*.* [12] found a closer relationship between Landrace and Yorkshire and a more distant relationship between Duroc and Landrace/Large. By studying genetic diversity in native and commercial pig breeds in Portugal, including Duroc, Landrace, Large White and Pietrain, Vicente et al. [30] concluded that Duroc is the more distant breed relative to the others. This could explain why the lowest correlations were observed between SL1 and the other lines.

Reference populations must be large for accurate prediction in GEBV, and the use of a combined reference population would be desirable. However, the correlation of phase across pure lines was low, suggesting the need for a SNP panel with a higher density than the 60K panel, even when combining SL2 and SL3 or SL2 and DL2, which presented the highest correlation of phase across the pure lines (>0.9 for markers at distances up to 50 Kb).

The utilization of multibreed reference panels has been studied as a method to increase the reference population size [5,6,10]. Hayes et al. [5] indicated that multi-breed reference populations will be a valuable resource to fine mapping of QTL. de Roos et al. [10] concluded that multi-breed reference panels could increase the reliability of the GEBV when at least some animals of the target breed are included, and the benefit of combining populations increased when the populations have diverged for fewer generations. In addition, Daetwyler et al. [6] showed that GEBV are more accurate than pedigree-based BLUP, using a multibreed sheep training population. According to Daetwyler et al. [9], across breed accuracy depends on the LD between markers and QTL because the impact of the relatedness between the breeds is expected to be minimal. Thus, persistence of phase studies provide information for shaping multibreed, or in the case of the pig industry, *multiline* reference panels. Knowing the persistence of phase allows us to identify the lines that have diverged more recently and would provide higher relationship between reference and validation populations, a factor that plays a large role in the accuracy of the predictions.

## Conclusions

This work evaluated the persistence of LD and LD decay of pure and crossbred pig lines using real data, and by representing the crossbreeding structure of pig production. Our data demonstrated the potential of crossbreds as reference panels for purebred selection and also pinpointed the pure lines that could be combined in a *multiline* training population. This study proposed an equality of LD decay curves to evaluate significant differences regarding LD decay. Useful LD (>0.3) seems to extend over larger distances in pigs than in Holstein cattle, which implied that less dense SNP panels are needed in GS and GWAS in pigs. However, if *multiline* reference populations are used for GS, the required density of SNP panels should be higher compared with a single breed reference population.

## Methods

The data used for this study were obtained as part of routine data recording in a commercial breeding program. Samples collected for DNA extraction were only used for routine diagnostic purpose of the breeding program. Data recording and sample collection were conducted strictly in line with the Dutch law on the protection of animals.

### Data

The data for this study were obtained from animals from five pig pure lines (SL1, n = 1,307; SL2, n = 643; SL3, n = 276; DL1, n = 626; DL2, n = 1013), one F_1_ cross (DLF1, n = 186) and two commercial finishing crosses (TER1, n = 286; TER2, n = 330). SL1 and SL2 are synthetic sire lines; SL1 is a combination of Duroc (mostly) and Belgian Landrace created in about 1980. SL2 is a combination of Large White and Pietrain created in about 1975. SL3 is a Pietrain sire line. DL1 is Landrace based dam line and DL2 is a Large White based dam line. DLF1 is a commercial F_1_ cross resulting from crossing animals of DL1 and DL2. TER1 is a commercial finishing pig resulting from a cross between DLF1 and SL1. TER2 is also a commercial finishing pig that resulted from a cross between DLF1 and SL2. All pure lines were kept under strict inbreeding restrictions, with approximately 40 replacement sires per year and more than 250 gilt replacements per year.

Animals were genotyped using the Illumina Porcine SNP60 Beadchip, and all SNPs with an undefined position in Build 10.2 [31] were excluded, as well the SNPs on the X chromosome. The X chromosome recombines only in females; therefore, it was expected that the X chromosome would show higher LD than the overall genome [32], which could cause an overestimation of the LD. The R software [33] was used for within population marker quality control, using the package GenABLE [34]. Markers with a call rate <90%, MAF <0.05 and/or a *p*-value for the Hardy-Weinberg equilibrium <0.0001 were excluded. The summary of the quality control of genotype data is presented in [Additional file 1: Table S2].

To estimate the persistence of phase, the data were divided into four groups, according the description shown in Additional file 1: Table S3, and only SNPs that passed the quality control in all lines of each group were used. In group 1, the F_1_ (DLF1) cross was compared with its parental lines, while in groups 2 and 3 the finishing crosses (TER1 and TER2) were compared with their parental and grandparental lines. In group 4, which included only pure lines, each line was compared with all other pure lines.

### LD

For each pig line, the LD between SNPs was computed as the correlation of gene frequencies $\left({r}_{ij}^2\right)$ [35] using the function LD of the package genetics [36] of the software R [33]:

$$ {r}_{ij}^2 = \frac{{\left({p}_{ij} - {p}_i{p}_j\right)}^2}{p_i\left(1 - {p}_i\right){p}_j\left(1 - {p}_j\right)} $$

where *p*_*i*_ and *p*_*j*_ are the marginal allelic frequencies at the *i*^*th*^ and *j*^*th*^ SNP, respectively, and *p*_*ij*_ is the probability of the marker allele pair ij, which is estimated using maximum likelihood because genotype data were used [36].

### LD decay

Decay of LD with the distance between markers was compared between lines. Only SNPs that passed the quality control filtering in all lines were used in this analysis. The comparison was conducted by adjusting the nonlinear regression model proposed by Sved [15] to allow for testing a curve equality hypothesis [37] across the eight populations evaluated. For the curve equality test, the nonlinear model receives a dummy variable that represents each one of the eight populations. This complete model is described as:

1 $$ L{D}_{\mathrm{ik}}={\displaystyle \sum_{\mathrm{k}=1}^8{\mathrm{D}}_{\mathrm{k}}\left[\frac{1}{{}^{1 + 4{\beta}_k{d}_i}}\right]}+{\mathrm{e}}_{\mathrm{ik}}, $$

where:

*LD*_ik_ is the observed ${r}_{ij}^2$ for marker pair *i* of line *k*;

$$ {\mathrm{D}}_{\mathrm{k}}\ \mathrm{is}\ \mathrm{an}\ \mathrm{dummy}\ \mathrm{variable},\ \mathrm{such}\ \mathrm{t}\mathrm{hat}:\ {\mathrm{D}}_{\mathrm{k}}=\left\{\begin{array}{l}1\ \mathrm{if}\ \mathrm{t}\mathrm{he}\ \mathrm{o}\mathrm{bservation}\ {\mathrm{LD}}_{\mathrm{ik}}\ \mathrm{belong}\ \mathrm{t}\mathrm{o}\ \mathrm{t}\mathrm{he}\ \mathrm{group}\ \mathrm{k}\\ {}0\ \mathrm{o}\mathrm{t}\mathrm{he}\mathrm{rwise}\end{array}\right.\kern0.1em ; $$

*d*_*i*_ is the distance in Kb for marker pair *i*;

*β*_*k*_ is the coefficient that describes the decline of LD with distance for line *k*;

*e*_*ik*_ is a random residual, *e*_*ik*_ ~ *N*(0, *σ*^2^);

The complete model is adjusted to test the hypothesis that the same model can describe the LD decay of all lines:

$$ {H}_0^{(1)}:{\upbeta}_k=\upbeta \kern0.5em \forall\ k\ vs\kern0.5em {H}_a^{(1)}:\ {\upbeta}_k\ne\ \upbeta\ \mathrm{f}\mathrm{o}\mathrm{r}\ \mathrm{at}\ \mathrm{least}\ \mathrm{o}\mathrm{ne}\ {\upbeta}_k, $$

To test The ${H}_0^{(1)}$ hypothesis, the following comparison scheme was conducted, considering the complete (1) and the reduced (2) models:

2 $$ L{D}_{\mathrm{ik}}={\displaystyle \sum_{\mathrm{k}=1}^8{\mathrm{D}}_{\mathrm{k}}\left[\frac{1}{{}^{1 + 4\beta {d}_i}}\right]} + {\mathrm{e}}_{\mathrm{ik}}, $$

where a single parameter *β* for all lines is assumed.

The residual sum of squares of the complete (SQR_Ω_) and reduced (SQR_ω_) models are used to perform a chi-squared statistic: ${\chi}_{\mathrm{computed}}^2=\mathrm{N} \ln \left(\mathrm{S}\mathrm{Q}{\mathrm{R}}_{\Omega}/\mathrm{SQR}\upomega \right),$ in which N is the number of observed measures of LD. The hypothesis ${\mathrm{H}}_0^{(1)}$ is rejected if ${\chi}_{\mathrm{computed}}^2\ge {\chi}_{\alpha (V)}^2,$ where ν = p_Ω_ − p_ω_ is the degree of freedom, where p_Ω_ and p_ω_ are the number of parameters of the complete and reduced models, respectively, at a significance level α.

Rejection of the hypothesis ${\mathrm{H}}_0^{(1)}$ implied that at least one parameter β differs from the others, and, subsequently, a pairwise comparison was carried out to identify the lines that are equal or different in relation to the parameter β. Multiple tests were carried out; therefore, the Bonferroni correction was employed to reduce Type I errors. In this case, the significance threshold (α*) was obtained by dividing the established significance threshold for a single test (α = 0.05) by the number of independent tests (n). Thus, for the present study, the significance level for pairwise comparison was α* = 0.05/28 = 0.0018.

The nonlinear models were adjusted using the function *nls* of the software R [33], and the hypothesis tests were also conducted using R scripts.

### Persistence of phase

The squared root of *r*_*ij*_ was obtained and given the same sign as D, which was calculated as described by Roos et al. [10], using the R software [33].

$$ D = {f}_{22}-\left({f}_{12} + {f}_{22}\right)\left({f}_{21}+{f}_{22}\right) $$

where:

$$ \begin{array}{c}\hfill {f}_{22}=\left(2{p}_{A22B22} + {p}_{A22B12} + {p}_{A12B22}\right)/\tau \hfill \\ {}\hfill {f}_{12}=\left(2{p}_{A11B22} + {p}_{A11B12} + {p}_{A12B22}\right)/\tau \hfill \\ {}\hfill {f}_{21}=2{p}_{A22B11} + {p}_{A22B12} + {p}_{A12B11}/\tau \hfill \\ {}\hfill \tau =2-2{p}_{A12B12}\hfill \end{array} $$

where *p*_*A*12*B*12_ is the proportion of animals with heterozygous genotypes at both loci.

This approach was first described by Goddard [38], and the setting of the D sign was conducted to consistently define the statistic in all lines. The *r*_*ij*_ received the same sign in two breeds if the same haplotype was more common than expected from the allele frequencies in both breeds.

To express the correlation of *r*_*ij*_ across populations in relation to the physical distances between SNPs, the Pearson correlations between *r*_*ij*_ values were calculated across lines for intervals of 50 kb (from 0 to 5000 kb). The interval of 50 kb was chosen based on the coefficient of variation (CV) of the number of SNP pairs for intervals of 10, 30, 50, 70 and 100 kb [see Additional file 1: Table S4] to guarantee that the most similar number of observations in each bin were used to calculate the correlation. Based on the CV evaluation, there was no evidence of difference in the use of bins of 30, 50, 70 and 100 kb; thus the value of 50 Kb was chosen to give a more detailed LD description in relation to the bins of 70 and 100 kb, and a better visualization in relation to the bin of 30 kb.

## Additional file

Additional file 1: Table S1. Values of $$ \begin{array}{l}{\chi}_{\mathrm{computed}}^2\\ {}\end{array} $$ (below the diagonal) and p-values for the pairwise comparison. **Table S2** SNP data description according to the quality control criteria. **Table S3** Grouping of lines and the number of SNPs for persistence of phase estimation. **Table S4** Coefficient of variation (CV) for the number of SNP pairs in bins of 10, 30, 50, 70 and 100 Kb.
