# Supplementary material for: Linkage disequilibrium patterns and persistence of phase in purebred and crossbred pig (Sus scrofa) populations
Source: BMC Genet. 2014 Nov 25;15:126. doi: 10.1186/s12863-014-0126-3 (PMC4261888; doi:10.1186/s12863-014-0126-3)
Supplement: Additional file 1: Table S1. — Values of $\begin{array}{l}{\chi}_{\mathrm{computed}}^2\\ {}\end{array}$χcomputed2 (below the diagonal) and p-values for the pairwise comparison. Table S2 SNP data description according to the quality control criteria. Table S3 Grouping of lines and the number of SNPs for persistence of phase estimation. Table S4 Coefficient of variation (CV) for the number of SNP pairs in bins of 10, 30, 50, 70 and 100 Kb. [file 12863_2014_126_MOESM1_ESM.docx]

**Additional files**

## Additional file 1. Supplementary tables.

**Table S1.** Values of $\begin{matrix} \chi_{computed}^{2} \\ \end{matrix}$(below the diagonal) and p-values for the pairwise comparison

|  | $\hat{\beta}_{SL1}$ | $\hat{\beta}_{SL2}$ | $\hat{\beta}_{SL3}$ | $\hat{\beta}_{DL1}$ | $\hat{\beta}_{DL2}$ | $\hat{\beta}_{DLF1}$ | $\hat{\beta}_{TER1}$ | $\hat{\beta}_{TER2}$ |
| --- | --- | --- | --- | --- | --- | --- | --- | --- |
| $\hat{\beta}_{SL1}$ | - | <10^-6^ | <10^-6^ | <10^-6^ | <10^-6^ | <10^-6^ | <10^-6^ | <10^-6^ |
| $\hat{\beta}_{SL2}$ | 6346.2 | - | <10^-6^ | <10^-6^ | <10^-6^ | <10^-6^ | <10^-6^ | <10^-6^ |
| $\hat{\beta}_{SL3}$ | 130.96 | 4663.36 | - | <10^-6^ | 0.0117* | <10^-6^ | <10^-6^ | <10^-6^ |
| $\hat{\beta}_{DL1}$ | 1380.75 | 13782.9 | 2374.35 | - | <10^-6^ | <10^-6^ | <10^-6^ | <10^-6^ |
| $\hat{\beta}_{DL2}$ | 81.72 | 5108.87 | 6.35 | 2179.95 | - | <10^-6^ | <10^-6^ | <10^-6^ |
| $\hat{\beta}_{DLF1}$ | 4561.1 | 22155.14 | 6290.32 | 902.39 | 6017.03 | - | <10^-6^ | <10^-6^ |
| $\hat{\beta}_{TER1}$ | 10848.53 | 12284.15 | 13452.27 | 4481.85 | 13143.96 | 1412.85 | - | <10^-6^ |
| $\hat{\beta}_{TER2}$ | 845.92 | 12284.15 | 1670.97 | 79.69 | 1498.69 | 1585.25 | 6031.08 | - |

Using Bonferroni correction: $\alpha^{*}=0.0018$

**Table S2.** SNP data description according to the quality control criteria

|  | SL1 | SL2 | SL3 | DL1 | DL2 | DLF1 | TER1 | TER2 |
| --- | --- | --- | --- | --- | --- | --- | --- | --- |
| MAF < 0.05 | 7,969 | 9,550 | 6,627 | 6,399 | 7,271 | 4,262 | 2,705 | 4,685 |
| HWE P-value <0.0001 | 478 | 2,500 | 1,535 | 4,999 | 1,526 | 2,837 | 5,239 | 3,493 |
| SNP call rate < 90% | 581 | 1,543 | 4,075 | 1,366 | 1,629 | 2,334 | 1,050 | 1,279 |
| SNPs utilized | 38,769 | 35,505 | 36,136 | 35,392 | 38,058 | 38,529 | 38,752 | 38,583 |
| Number of animals | 1,307 | 643 | 276 | 626 | 1,013 | 186 | 286 | 330 |

**Table S3.** Grouping of lines and the number of SNPs for persistence of phase estimation

| Lines | Group 1 | Group 2 | Group 3 | Group 4 |
| --- | --- | --- | --- | --- |
| DL1 | X | X | X | X |
| DL2 | X | X | X | X |
| SL1 |  | X |  | X |
| SL2 |  |  | X | X |
| SL3 |  |  |  | X |
| DLF1 | X | X | X |  |
| TER1 |  | X |  |  |
| TER2 |  |  | X |  |
| Number of SNPs | 28,153 | 22,272 | 22,794 | 20,435 |

**Table S4.** Coefficient of variation (CV) for the number of SNP pairs in bins of 10, 30, 50, 70 and 100 Kb

| Distance intervals (Kb) | Group1 | Group2 | Group3 | Group4 |
| --- | --- | --- | --- | --- |
| 10 | 0.066 | 0.078 | 0.077 | 0.075 |
| 30 | 0.056 | 0.068 | 0.067 | 0.064 |
| 50 | 0.055 | 0.068 | 0.066 | 0.063 |
| 70 | 0.055 | 0.067 | 0.066 | 0.063 |
| 100 | 0.055 | 0.068 | 0.066 | 0.063 |
